# Efficacy of Adding Bevacizumab in the First-Line Chemotherapy of Metastatic Colorectal Cancer: Evidence from Seven Randomized Clinical Trials

**DOI:** 10.1155/2014/594930

**Published:** 2014-05-25

**Authors:** Yan-xian Chen, Qiong Yang, Jun-jie Kuang, Shi-yu Chen, Ying Wei, Zhi-min Jiang, De-rong Xie

**Affiliations:** ^1^Department of Oncology, Sun Yat-sen Memorial Hospital, Sun Yat-sen University, Guangzhou, Guangdong 510515, China; ^2^Department of Internal Medicine, Nanfang Hospital of Southern Medical University, Luohu District, Guangdong 510515, China

## Abstract

*Background*. Efficacy of adding bevacizumab in first-line chemotherapy of metastatic colorectal cancer (mCRC) has been controversial. The aim of this study is to gather current data to analyze efficacy of adding bevacizumab to the most used combination first-line chemotherapy in mCRC, based on the 2012 meta-analysis reported by Macedo et al.  *Methods*. Medline, EMBASE and Cochrane library, meeting presentations and abstracts were searched. Eligible studies were randomized controlled trials (RCTs) which evaluated first-line chemotherapy with or without bevacizumab in mCRC. The extracting data were included and examined in the meta-analysis according to the type of chemotherapy regimen. *Results*. Seven trials, totaling 3436 patients, were analyzed. Compared with first-line chemothery alone, the adding of bevacizumab did not show clinical benefit for OS both in first-line therapy and the most used combination chemotherapy (HR = 0.89; 95% CI = 0.78–1.02; *P* = 0.08; HR = 0.93; 95% CI = 0.83–1.05; *P* = 0.24). In contrast with OS, the addition of bevacizumab resulted in significant improvement for PFS (HR = 0.68; 95% CI = 0.59–0.78; *P* < 0.00001). Moreover, it also demonstrated statistical benefit for PFS in the most used combination first-line chemotherapy (HR = 0.84; 95% CI = 0.75–0.94; *P* = 0.002). And the subgroup analysis indicated only capacitabine-based regimens were beneficial. *Conclusions*. This meta-analysis shows that the addition of bevacizumab to FOLFOX/FOLFIRI/XELOX regimens might not be beneficial in terms of OS. Benefit has been seen when PFS has been taken into account. In subgroup analysis, benefit adding bevacizumab has been seen when capecitabine-based regimens are used. Further studies are warranted to explore the combination with bevacizumab.

## 1. Introduction

Colorectal cancer is the second most frequent cause of death in Europe and North America with about 600,000 deaths and a rate of approximately 1.2 million new cases every year in the whole world [1]. In order to improve the survival, more and more drugs have been introduced to the treatments of mCRC, including novel targeted drugs.

Bevacizumab, a humanized monoclonal antibody, binds to vascular endothelial growth factor (VEGF-) A, and inhibits signaling through the VEGF receptors [2]. It was approved by US Food And Drug Administration (FDA) in 2004, based on the AVF2107 study [3] comparing bolus irinotecan, 5 fluorouracil, leucovorin (IFL), and placebo therapy with IFL and bevacizumab showed a 4.7 and 4.4 month increase in median OS and PFS, respectively. However, FOLFOX, FOLFIRI [4, 5], and XELOX [6–8] became standard first-line therapy for mCRC now. Comparing FOLFOX regimen, IFL showed more early deaths, greater toxicity, and worse effectiveness in N9741 study [9]. So we explore the idea of bevacizumab added to the most used combination chemotherapy regimens—FOLFOX, FOLFIRI, and XELOX—being as beneficial as its addition to IFL regimen, as in the AVF2107 trial [3].

Macedo et al. [10] carried out a meta-analysis about addition of bevacizumab to first-line chemotherapy in advanced colorectal cancer, which indicated bevacizumab shows a survival benefit in mCRC, but the current data was insufficient to support efficacy in all regimens, especially infusional fluorouracil regimens, like FOLFIRI and FOLFOX. Recently, the ITACA study, reported in ASCO meeting [11], demonstrated no statistically significant differences in PFS, OS, and ORR for adding bevacizumab to the first-line chemotherapy of mCRC.

With the advent of new trials, this meta-analysis tried to gather current data, including six RCTs from Macedo et al. meta-analysis [10] and ITACA trial [11] and then analyzed efficacy of adding bevacizumab to first-line chemotherapy or the most used combination chemotherapy of mCRC, compared with chemotherapy alone.

## 2. Methods 

### 2.1. Search Strategy

The search strategy is similar to the one in the 2012 meta-analysis [10]. Articles published or presented from March 2011 to June 2013 were searched in the electronic databases including MEDLINE, EMBASE, and The Cochrane Library. Conference websites from ASCO, ESMO, and the World Congress on Gastrointestinal Cancer from 2011 to 2013 were also examined. An effective search strategy was performed through terms related to colorectal neoplasms, bevacizumab, and randomized controlled trials in all fields. Language was limited in English. For PubMed we used the search strategy as follows: (“Colorectal Neoplasms” [Mesh] AND “bevacizumab” [Supplementary Concept]) AND “Randomized Controlled Trials as Topic” [Mesh]. The deadline of this search was on 30 June 2013. At last, 127 potentially eligible abstracts were collected.

### 2.2. Selection Criteria and Quality Assessment

The studies must satisfy randomized, controlled clinical trials with a prospective design comparing first-line chemotherapy adding bevacizumab with chemotherapy alone for advanced colorectal cancer, which were previously untreated or without chemotherapy for 6 months, in case of adjuvant therapy. Those studies of poor quality (Jadad score < 3 [17]) or involving other targeted drugs were excluded. It also should be excluded if data was not available. Methodological quality [17] of the trials was evaluated by Jadad score (range from 0 to 5) which contained randomization, masking, dropouts, and withdrawals.

### 2.3. Data Extraction

The main measurement outcome was OS (time from random assignment to death). The second was PFS (the time from random assignment to the first documentation of progression for disease, or death from any cause). HR and its 95% confidence intervals (CI) of OS and PFS were directly collected from each selected study. When the trial had two comparative regimens, if the total HR existed, it was preferentially extracted; otherwise, the HR of each arm was selected. If data of the six primary studies reported were insufficient, they were referenced from the data shown in 2012 meta-analysis [10] which contacted corresponding authors with a request for the pending information. The following information was also extracted from each published trial: year of publication, first author, number of patients, chemotherapy regimen, follow-up period, and so forth.

The relevant articles identified from the above search strategy were assessed by two independent reviewers (Yan-xian Chen and Qiong Yang). Disagreements were dealt with to achieve consensus or by a third reviewer (De-rong Xie).

### 2.4. Statistical Analysis and Synthesis

Statistical heterogeneity was assessed through chi-square and *I*-square test. If heterogeneity was detected (*P* value < 0.1 or *I* ^2^ > 50%), using random model, a possible explanation for it was performed. When a reasonable cause was found, the subgroup analysis was computed. If heterogeneity was not detected, otherwise, use fixed model. OS and PFS analyses were performed using a random or fixed effects analysis model and the generic inverse variance. The results calculated were expressed by the forest plots. The effect of the treatment of each single study was expressed as a ratio of the bevacizumab-added chemotherapy regiment over chemotherapy alone. A HR value of less than one implied a clinical benefit for association of bevacizumab. A *P* value less than 0.05 for Begg's test [18] was defined as publication bias. Meta-analyses for this study were conducted with RevMan 5.2 [19] and STATA version 12.0.

## 3. Results 

### 3.1. Search Results

**Figure 1:**
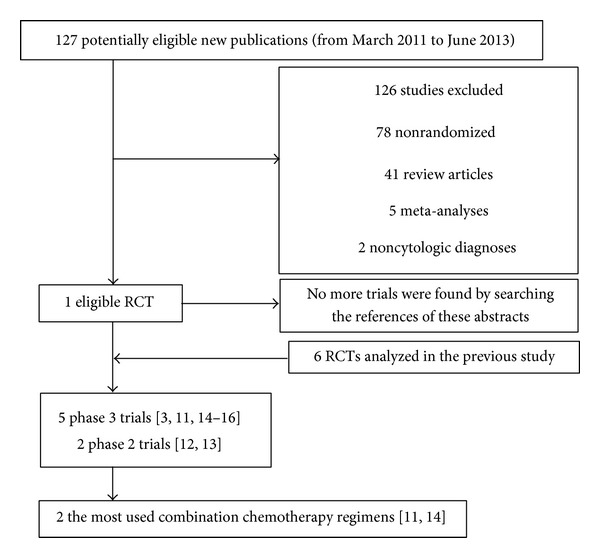
The flow chart.

1 new trial satisfying the above conditions was found. There were a total of 7 selected articles, when added to the previous 6 trials extracted from the 2012 meta-analysis reported by Macedo et al. [3, 11–16]. Only NO16966 study and ITACA trial contained the most used combination chemotherapy regimens. Two reviewers finally made an agreement to select these trials including 3436 patients in the meta-analysis. The flow chart is presented in Figure 1.  Table 1 describes the main details of the extracted studies.

### 3.2. Overall Survival

**Figure 2:**
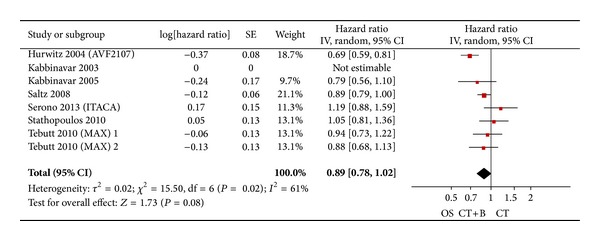
Randomized effect model on HR of OS in first-line chemotherapy. The pooled HR of OS in first-line chemotherapy is symbolized by a solid diamond at the bottom of the forest plot and the width of which represents the 95% CI.

Regarding OS, six studies accessed this outcome, enrolling 3314 patients. The result of the test for heterogeneity of the therapeutic effect was significant (*P* = 0.02). A random effect model was adopted. In first-line bevacizumab-added chemotherapy, compared to chemotherapy alone, the meta-analysis of data did not reach statistical significance for OS (HR = 0.89; 95% CI = 0.78–1.02; *P* = 0.08) (Figure 2).

**Figure 3:**
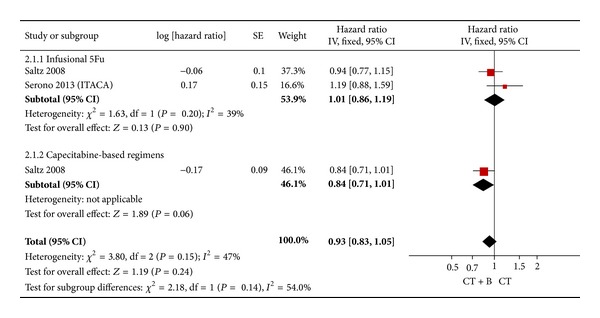
Fixed effect model on HR of OS in the most used combination chemotherapy. The pooled HR of OS in standard first-line chemotherapy is symbolized by a solid diamond at the bottom of the forest plot and the width of which represents the 95% CI.

We further analyzed the efficacy of adding bevacizumab in the most used combination chemotherapy. In these seven trials, only NO16966 study and ITACA trial included the most used combination chemotherapy regimens, enrolling 1777 patients. The result of the test for heterogeneity of the treatment effect was not significant (*P* = 0.15). Therefore, a fixed effects model was selected in meta-analysis. The addition of bevacizumab did not demonstrate favorable results (HR = 0.93; 95% CI = 0.83–1.05; *P* = 0.24) (Figure 3). Subgroup analysis by the type of fluoropyrimidine administration also did not show statistically significant data (Figure 3).

### 3.3. Progression-Free Survival

**Figure 4:**
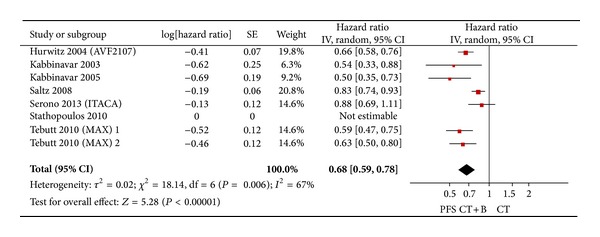
Randomized effect model on HR of PFS in first-line chemotherapy. The beneficial HR of PFS in standard first-line chemotherapy is symbolized by a solid diamond at the bottom of the forest plot and the width of which represents the 95% CI.

From the seven studies, one study did not offer related data. The results demonstrated an advantage to the association of bevacizumab using a random effect model (HR = 0.68; 95% CI = 0.59–0.78; *P* < 0.00001) compared to chemotherapy alone, though heterogeneity between trials was detected (*I* ^2^ = 67%; *P* = 0.006) (Figure 4).

**Figure 5:**
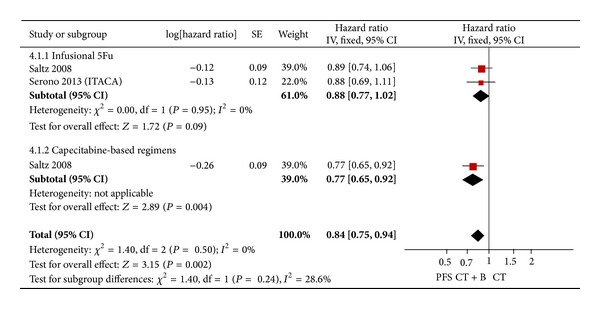
Fixed effect model on HR of PFS in the most used combination chemotherapy. The beneficial HR of PFS in standard first-line chemotherapy is symbolized by a solid diamond at the bottom of the forest plot and the width of which represents the 95% CI.

In the most used combination chemotherapy, the result of the test for heterogeneity of the treatment effect was not significant (*P* = 0.24). So, a fixed effects model was selected in meta-analysis. The analysis of the most used combination chemotherapy plus bevacizumab versus chemotherapy alone indicated uniform benefit (HR = 0.84; 95% CI = 0.75–0.94; *P* = 0.002). The subgroup analysis showed that only capecitabine-based regimens were beneficial (HR = 0.77; 95% CI = 0.65–0.92; *P* = 0.004) (Figure 5).

### 3.4. Publication Bias

**Figure 6:**
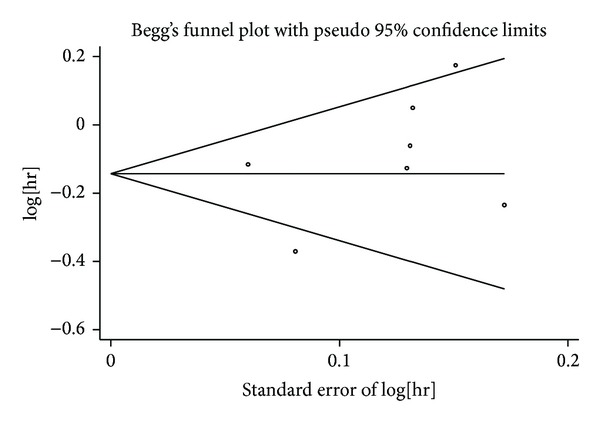
Funnel plot for publication bias test OS. The two oblique lines indicate the pseudo 95% confidence limits.

**Figure 7:**
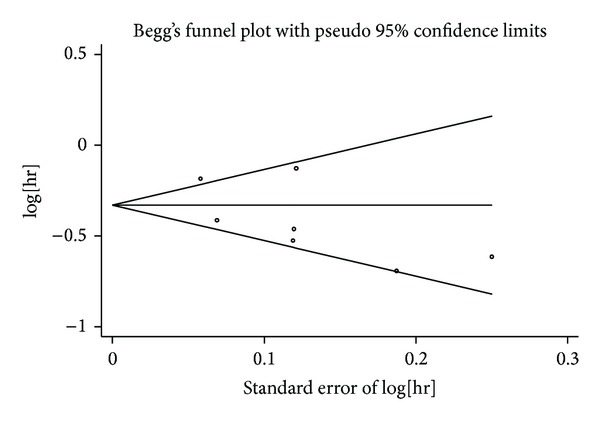
Funnel plot for publication bias test PFS. The two oblique lines indicate the pseudo 95% confidence limits.

The results still did not suggest any evidence of publication bias according to the shape of funnel plots (Figures 6 and 7) or Begg's test in OS (*z* = 1.05, *P* = 0.293) and PFS (*z* = −0.75, *P* = 0.453).

## 4. Discussion

According to our meta-analysis, the addition of bevacizumab to first-line therapy does not prolong overall survival. HR value of the most used combination chemotherapy with adding bevacizumab rises from 0.89 to 0.93, which has a much narrower 95% confidence interval (from 95% CI = 0.78–1.02 to 95% CI = 0.83–1.05) and better homogeneity (from *I* ^2^ = 61%, *P* = 0.02 to *I* ^2^ = 47%; *P* = 0.15), suggesting the result of no survival benefit regards to OS is more powerful.

Regarding PFS, however, it appears to be advantageous in both first-line therapy and the most used combination first-line chemotherapy. To further analyze, it is the XELOX subgroup that shows the benefit, supported by the single-study NO16966 [14]. In contrast to the XELOX subset, infusional 5-Fu regimens, like FOLFOX or FOLFIRI, do not prolong PFS. The administration and the drug type of fluoropyrimidine might be the main reason for the distinct therapeutic effect of bevacizumab addition to different fluoropyrimidine-based chemotherapy regimens.

The addition of bevacizumab to first-line chemotherapy in mCRC is still a paradigm left unsolved. Compared with 2012 meta-analysis, adding the ITACA study [11] which only takes the weight of 11.3% in all enrolled trials, the entire consequence of this meta-analysis is quite different regarding OS. It means that a survival benefit of the 2012 meta-analysis is not obvious, and the conclusion is very unsound. But for PFS, the result is quite similar. Addition of bevacizumab to infusional 5-Fu-based regimens, like FOLFOX or FOLFIRI, does not show clinical benefit, while adding bevacizumab to capacitabine-based regimens are beneficial. It may be instrumental to find which chemotherapy regimen is effective associated with bevacizumab by comparing the clinical benefit between bevacizumab and unhomogeneous fluorouracil agents.

The benefit of PFS cannot turn into OS so far according to the above results. Firstly, of the seven selected trials, three made PFS as primary endpoint for original design, especial NO16966 study [14] and ITACA trial [11]. However, we choose OS as the main measurement outcome in this meta-analysis. Thus, the distinct experimental design may be the reason why the benefit of PFS cannot turn into OS. In addition, the current data proves that bevacizumab plus chemotherapy (crossed over from first-line regimen) which continued beyond progression significantly prolonged OS and PFS (1.4 months, 1.6 months, resp.) in second-line mCRC in ML18147 study [20]. But, the treatment of combination with bevacizumab is limited to the first-line in all enrolled trials, without considering the effect of the second- and further-line regimen on OS. Moreover, preliminary data suggests that there are some groups that might benefit more from the combination of bevacizumab, but the included studies of our meta-analysis cannot explore these factors, just like patients with primary tumors originating in the rectum and sigmoid colon [21], or certain targets like wild-type KRAS [22] and mismatch repair defective (dMMR) tumors [23], or the combination with certain drugs for medication interactions. Further studies are required to confirm the findings.

Several limitations have to be mentioned regarding this meta-analysis. One is that all of the data are extracted from abstracted data (AD) and not individual patient data (IPD), which would be less convincing to confirm the results. But, a correlation analysis shows AD meta-analysis is strongly associated with IPD meta-analysis [24]. It means AD is a kind of acceptable and practical method of meta-analysis alternative for IPD. Another limitation is that we chose OS as the main endpoint instead of PFS which was the primary endpoint originally in most trials. As we all know, OS is a more objective index and an ending point in the field of incurable diseases like mCRC, and it needs to enroll more cases in the trial. However PFS may be influenced by the sensitivity of imaging instrument, experience of checker, and timeliness discovery of the tumor progression. In addition, the studies enroll 1777 patients in the most used combination chemotherapy regimens. They are too few to carry out more subgroups analysis. Only ITACA study contained FOLFIRI regimen. However, it cannot perform subsets analysis because we cannot obtain more information about bevacizumab addition to FOLFIRI or FOLFOX regimen from the ITACA abstract reported in the 2013 ASCO meeting.

## 5. Conclusion 

This meta-analysis shows that the addition of bevacizumab to FOLFOX/FOLFIRI/XELOX regimens might not be beneficial in terms of OS, compared to chemotherapy alone. Benefit has been seen when PFS has been taken into account. In subgroup analysis, benefit of adding bevacizumab has been seen when capecitabine-based regimens are used. Further studies are warranted to explore the combination with bevacizumab.

## Figures and Tables

**Table 1:** Characteristics of trials about the first-line therapy.

Studies	Intervention	Patients	Median follow-up (mo)	Median OS and 95% CI (mo)	HR and 95% CI for OS	Median PFS and 95% CI (mo)	HR and 95% CI for PFS	Jadad score
Hurwitz et al. 2004 (AVF2107) [3]	IFL + B	402	Not reported	20.3	0.69 [0.59, 0.81]	10.6	0.66 [0.58, 0.76]	4
	IFL	411		15.6^∧^		6.2^∧^		

Kabbinavar et al. 2003 [12]	Fu + LV + Bh	33	Not reported	16.1 [11.0–20.7]	Not reported	7.2 [3.8–9.2]	0.54 [0.33, 0.88]	3
	Fu + LV + Bl	35		21.5 [17.3-undetermined]		9.0 [5.8–10.9]		
	Fu + LV	36		13.8 [9.1–23.0]		5.2 [3.5–5.6]		

Kabbinavar et al. 2005 [13]	Fu + LV + B	104	Not reported	16.6	0.79 [0.56, 1.10]	9.2	0.50 [0.34, 0.73]	4
	Fu + LV	105		12.9^∧^		5.5^∧^		

Saltz et al. 2008 (N016966)* [14]	XELOX + B	350	27.6	Not reported	0.84 [0.71, 1.01]	Not reported	0.77 [0.65, 0.92]	4
	XELOX	350		Not reported		Not reported		
	FOLFOX-4 + B	350	27.6	Not reported	0.94 [0.77, 1.15]	Not reported	0.89 [0.74, 1.06]	4
	FOLFOX-4	351		Not reported		Not reported		

ITACA* [11]	FOLFOX-4 or FOLFIRI + B	179	Unclear^#^	20.6 [15.3, 22.6]	1.18 [0.88, 1.58]	9.2 [8.0, 10.0]	0.88 [0.70, 1.10]	≥3
	FOLFOX-4 or FOLFIRI	197		20.6 [18.2, 23.3]		8.4 [7.0, 8.9]		

Stathopoulos et al. 2010 [15]	mIFLl + B	114	72	22.0 [18.1, 25.9]	1.05 [0.81, 1.36]	Not reported	Not reported	3
	mIFL	108		25.0 [18.1–31.9]		Not reported		

Tebbutt et al. 2010 (MAX) 2 [16]	CBM	158	21.5	16.4	0.94 [0.73, 1.21]	8.4 [7.5–9.0]	0.59 [0.47, 0.75]	3
	C	156		18.9^∧^		5.7 [5.4–6.2]		

Tebbutt et al. 2010 (MAX) 1 [16]	CB	157	48	Not reported	0.88 [0.68, 1.13]	8.5 [7.3–9.2]	0.63 [0.50, 0.79]	3
	C	156		Not reported		5.7 [5.4–6.2]		

*The most used combination chemotherapy in metastatic colorectal cancer. ^#^The data is unclear because it is just extracted from the abstract from 2013 ASCO annual meeting. ^∧^95% CI was not reported.

B: bevacizumab, Bl: bevacizumab in low doses, Bh: bevacizumab in high doses, XELOX: Xeloda plus oxaliplatin regimen, FOLFOX: 5-Fu, LV, and oxaliplatin regimen.

FOLFIRI: 5-Fu, LV, and irinotecan regimen, mIFL: modified irinotecan, LV, and 5-Fu regimen, CBM: capecitabine, bevacizumab, and mitomycin regimen.

CB: capecitabine and bevacizumab regimen, C: capecitabine.
